# Expanding the Universe of Hemoplasmas: Multi-Locus Sequencing Reveals Putative Novel Hemoplasmas in Lowland Tapirs (*Tapirus terrestris*), the Largest Land Mammals in Brazil

**DOI:** 10.3390/microorganisms10030614

**Published:** 2022-03-14

**Authors:** Anna Claudia Baumel Mongruel, Emília Patrícia Medici, Ariel da Costa Canena, Ana Cláudia Calchi, Rosangela Zacarias Machado, Marcos Rogério André

**Affiliations:** 1 Immunoparasitology Laboratory, Department of Pathology, Theriogenology, and One Health, School of Agricultural and Veterinary Sciences, São Paulo State University, UNESP, Jaboticabal 14884-900, SP, Brazil; annaclaudiamongruel@gmail.com (A.C.B.M.); ana.calchi@unesp.br (A.C.C.); rzacariasmachado@gmail.com (R.Z.M.); 2 Iniciativa Nacional para a Conservação da Anta Brasileira (INCAB), Instituto de Pesquisas Ecológicas (IPÊ), Campo Grande 79046-150, MS, Brazil; medici@ipe.org.br (E.P.M.); ariel.canena@gmail.com (A.d.C.C.); 3 Escola Superior de Conservação Ambiental e Sustentabilidade (ESCAS/IPÊ), Nazaré Paulista 12960-000, SP, Brazil; 4 Tapir Specialist Group (TSG), International Union for Conservation of Nature (IUCN SSC), Campo Grande 79046-150, MS, Brazil

**Keywords:** hemotropic *Mycoplasma*, lowland tapirs, Pantanal, Cerrado

## Abstract

The lowland tapir (*Tapirus terrestris*) is the largest land mammal in Brazil and classified as a vulnerable species, according to the assessment of the risk of extinction. The present study aimed at investigating the occurrence and genetic diversity of hemoplasmas in free-ranging *T. terrestris* from the Brazilian Pantanal and Cerrado biomes. Blood samples were collected from 94 living and eight road-killed tapirs, totalizing 125 samples Conventional PCR targeting four different genes (16S rRNA, 23S rRNA, *RNAse P*, and *dnaK*) were performed, and the obtained sequences were submitted for phylogenetic, genotype diversity, and distance analyses. The association between hemoplasma positivity and possible risk variables (age, gender, and origin) was assessed. Out of 122 analyzed samples, 41 (41/122; 33.61% CI: 25.84–42.38%) were positive in the 16S rRNA-based PCR assay for hemoplasmas. Positivity for hemoplasmas did not differ between tapirs’ gender and age. Tapirs from Pantanal were 5.64 times more likely to present positive results for hemoplasmas when compared to tapirs sampled in Cerrado. BLASTn, phylogenetic, genotype diversity, and distance analyses performed herein showed that the sampled lowland tapirs might be infected by two genetically distinct hemoplasmas, namely ‘*Candidatus* Mycoplasma haematoterrestris’ and ‘*Candidatus* Mycoplasma haematotapirus’. While the former was positioned into “*Mycoplasma haemofelis* group” and closely related to ‘*Candidatus* Mycoplasma haematoparvum, the latter was positioned into “*Mycoplasma suis* group” and closely related to ‘*Candidatus* Mycoplasma haematobos’. The impact of both putative novel species on tapir health status should be investigated.

## 1. Introduction

The lowland tapir (*Tapirus terrestris*) is the largest land mammal in Brazil and the species is widely distributed throughout the country. Although the lowland tapir can still be found in four Brazilian biomes (Atlantic Forest, Pantanal wetlands, Amazon, and Cerrado), this species is classified as vulnerable according to extinction risk assessments [1,2].

The emergence of infectious diseases in tapir populations may occur as a consequence of habitat loss and increased contact between animals. In this instance, preventive medicine should be applied for both in situ and ex situ conservation attempts [3]. In the last decades, studies focusing on direct detection of different pathogens, such as *Trypanosoma terrestris* [4,5], *Theileria* sp. [6,7], and *Mycobacterium* sp. [8] and also serological evidence for bacterial, viral [9,10], and rickettsial [11] exposure have been reported in tapirs from Brazil.

The *Mycoplasma* genus (Mollicutes: Mycoplasmataceae) comprises bacteria lacking a cell wall and presenting a small genome (under 1 Mbp) [12,13,14]. Belonging to the *Mycoplasma* genus, hemoplasmas (hemotropic mycoplasmas) encompass species that attach to erythrocytes’ cell membranes of different mammalian hosts [15]. Even though the consequences of hemoplasma infection are still unrevealed for many vertebrate species [16,17,18] it has been associated with anemia in cats infected by *Mycoplasma haemofelis* [19,20] and sheep infected by *Mycoplasma ovis* [21].

Cultivation in vitro has not been achieved for hemoplasmas up to now. For this reason, the diagnosis mainly relies on the molecular detection of selected gene fragments. Recently, newly described genotypes and *Candidatus* species have been reported in wild animals from Brazil based on PCR amplification of selected molecular markers, namely 16S rRNA and 23S rRNA genes. Potentially novel species have been identified in capybaras (*Hydrochoerus hydrochaeris*) [22] hairy dwarf porcupines (*Sphiggurus villosus*) [23], opossums (*Didelphis albiventris*) [24], and coatis (*Nasua nasua*) [25] from Brazil. Moreover, putative new genotypes have also been reported in free-ranging animals from different regions of the country, such as non-human primates from the Brazilian Amazon [26] rodents from several Brazilian biomes [27] and bats [28,29].

To the best of the authors’ knowledge, this is the first report of hemoplasma infection in lowland tapirs. The present study aimed to investigate the occurrence and genetic diversity of hemotropic *Mycoplasma* in free-ranging *T. terrestris* from the Pantanal and Cerrado biomes in Brazil.

## 2. Materials and Methods

### 2.1. Study Areas

The Cerrado biome is the second largest of the Brazilian biomes, after the Amazon. This biome spreads across 2 million km^2^ of the central Brazilian plateau and accounts for 21% of the country’s land area, representing the most extensive savannah region in South America and the one harboring the highest biodiversity worldwide. It consists largely of a mosaic of different ecosystems and vegetation types, including tree and scrub savannah, grassland with scattered trees, and occasional patches of a dry, closed-canopy forest called ‘Cerradão’. Gallery forests are found throughout the region along rivers and streams [30].

The Pantanal is the largest continuous freshwater wetland on the planet, encompassing 179,300 km^2^ of low elevation floodplains of the upper Paraguay River, in the center of South America (Brazil, 78%; Bolivia, 18%; Paraguay, 4%). Dry and wet seasons are well defined and rainfall concentrates between November to March, during the summer, favoring the occurrence of a seasonal flood pulse. The landscape is known to include open woodlands, forests, floodable, and nonfloodable grasslands, and also temporary or permanent aquatic habitats [31]. The Pantanal wetland is one of the most important strongholds for tapirs in South America as it holds a large, continuous, healthy tapir population [32].

Most of the Pantanal wetland is held in private lands (93% of the land on the Brazilian side) [31]. Baía das Pedras Ranch is a private property of 145 km^2^ in the Nhecolândia subregion of the Southern Pantanal, Municipality of Aquidauana, Mato Grosso do Sul State. The main activity is extensive cattle raising over native grasses, the traditional method. This ranch includes a mosaic of seasonally inundated grasslands, lakes, gallery forests, scrub, and deciduous forests that supports an abundance of wildlife which is also exploited through ecotourism by the owners.

Currently, Brazil is facing a historic scenario of natural areas destruction. In 2020, 23% of Pantanal were estimated to be burned out by wildfires [33]. In 2021, the Cerrado presented an increase of 7.9% in the suppression of natural vegetation when compared to the previous year, totaling ~8531.44 km^2^ [34]. It is estimated that wildfires may have affected at least 65 million native vertebrates and 4 billion invertebrates, including endangered species such as the jaguar (*Panthera onca*), giant anteater (*Myrmecophaga tridactyla*), marsh deer (*Blastocerus dichotomus*), crowned solitary eagle (*Buteogallus coronatus*), and hyacinth macaw (*Anodorhynchus hyacinthinus*). The impacts caused by wildfires are related as direct injuries and death, or indirect injuries caused by habitat loss and resource depletion [35].

### 2.2. Sampling

This study was approved by the Ethics Committee for Animal Experimentation of FCAV/UNESP (Faculty of Agricultural and Veterinary Sciences of the São Paulo State University) under protocol number 4558/20. The “Instituto Chico Mendes de Conservação da Biodiversidade (ICMBIO)” provided the required annual permits for the capture and immobilization of tapirs and collection of biological samples (SISBIO# 14,603). All protocols for the capture, anesthesia, handling, and sampling of tapirs have been reviewed and approved by the Veterinary Advisors of the Association of Zoos and Aquariums (AZA)—Tapir Taxon Advisory Group (TAG), and the Veterinary Committee of the IUCN SSC Tapir Specialist Group (TSG).

From 2013 to 2018, blood samples from free-ranging *T. terrestris* were collected from 94 living and 8 road-killed individuals, totalizing 125 samples. From this total, 78 (78/125; 62.40% CI: 53.66–70.40%) samples were collected from 61 tapirs (61/94; 64.89%; CI: 54.83–73.78%) in the Pantanal wetland and 39 samples (39/125; 31.20% CI: 23.74–39.78%) were collected from 33 tapirs (33/94; 35.11% 26.22–45.17%) from the Cerrado. Both study areas are located in Mato Grosso do Sul State, central-western Brazil. Sampling was performed during tapir anesthesia for the installation of GPS collars by professionals from the “Iniciativa Nacional para a Conservação da Anta Brasileira (INCAB-IPÊ)” (Lowland Tapir Conservation Initiative (LTCI-IPÊ)). Some living animals (*n* = 20) were sampled more than once. Moreover, eight samples (8/125; 6.40% CI: 3.28–12.12%) belonging to eight road-killed animals on highways BR-267 and MS-040 in the Cerrado biome were collected during necropsy procedures.

The sampled living animals included females (46/94; 48.94% CI: 39.07–58.88%), males (48/94; 51.06% CI: 41.12–60.93%), adults (>48 months old) (50/94; 53.19% CI: 43.18–62.95%), and sub-adults (<48 months old) (44/94; 46.81% CI: 37.05–56.82%). The road killed animals included females (3/8; 37.50% CI: 13.68–69.43%), males (5/8; 62.50% CI: 30.57–86.32%), adults (>48 months old) (5/8; 62.50% CI: 30.57–86.32%), and sub-adults (<48 months old) (3/8; 37.50% CI: 13.68–69.43%). In total, 125 biological samples were obtained from 102 individual tapirs (living or road killed) identified as females (49/102; 48.04% CI: 38.59–57.63), males (53/102; 51.96% CI: 42.37–61.41), adults (55/102; 53.92% CI: 44.28–63.28%), and sub-adults (47/102; 46.08% CI: 36.72–55.72%).

### 2.3. DNA Extraction and PCR Protocols for Mammals’ Endogenous Genes

DNA extraction was performed using a commercial kit (InstaGene™ Matrix, Biorad^®^, Hercules, CA, USA) and following the manufacturers’ instructions. In order to ensure successful DNA extraction, a conventional PCR for the mammal-endogenous gene glyceraldehyde-3-phosphate dehydrogenase (gapdh) targeting a 450 bp fragment [36] was performed in all samples. Samples that were not successfully amplified by the gapdh-based PCR protocol were submitted to a second protocol targeting a 227 bp fragment of the irpb (interphotoreceptor retinoid-binding protein) gene [37]. Samples that did not yield amplicons in either of the used PCR protocols were excluded from the subsequent analysis.

### 2.4. Conventional Polymerase Chain Reaction (cPCR) Assays for Hemoplasmas Based on the 16S rRNA, 23S rRNA, RNAse P and dnaK Gene Fragments

Samples were screened for the presence of hemoplasmas DNA using a semi-nested PCR protocol targeting a fragment of approximately 1107 bp of the 16S rRNA gene [38,39]. Positive samples were submitted to PCR protocols targeting the 23S rRNA (approximately 800 bp) [18], *RNAse P* (approximately 165 bp) [16], and *dnaK* (approximately 544 bp) genes [40] for additional molecular characterization. Thermal conditions and PCR reagent concentrations were slightly modified from the originally published protocols for *RNAse P* and *dnaK* genes (Table 1). DNA obtained from a naturally infected sheep with *Mycoplasma ovis* [18] and ultrapure RNAse and DNAse-free water (Promega, Madison, WI, USA) were used as positive and negative controls, respectively, in all PCR assays for hemoplasmas.

The products obtained in PCR assays were separated by electrophoresis on a 1% agarose gel stained with ethidium bromide (Life Technologies™, Carlsbad, CA, USA) at 100 V/150 mA for 50 min. The gels were imaged under ultraviolet light (ChemiDoc MP Imaging System, Bio Rad™, Hercules, CA, USA) using the Image Lab Software v4.1 (Biorad, Hercules, CA, USA).

### 2.5. Sequencing

Amplified products were purified using a commercial kit (Wizard^®^ SV Gel and PCR Clean-Up System, Promega, Madison, WI, USA) and sequenced using the BigDye™ Terminator v3.1 Cycle Sequencing Kit (Thermo Fisher Scientific™, Waltham, MA, USA) and ABI PRISM 310DNA Analyzer (Applied Biosystems™, Foster City, CA, USA) [41].

### 2.6. Sequence Analysis and Phylogeny

The obtained sequences were first submitted to a screening test using Bioedit v7.0.5.3 (http://www.mbio.ncsu.edu/BioEdit/bioedit.html, accessed on 8 December 2021) [42] to evaluate the electropherogram quality and generate the consensus sequences. The BLASTn program (National Center for Biotechnology Information, Bethesda, MD, USA) [43] was used to analyze the nucleotide sequences (BLASTn), aiming to browse and compare with sequences from an international database (GenBank) [44]. The consensus sequences obtained in the current study and those retrieved from GenBank were aligned using the ClustalW software [45] via Bioedit v7.0.5.3 (http://www.mbio.ncsu.edu/BioEdit/bioedit.html, accessed on 8 December 2021) [42] and also improved by an MAFFT alignment performed using GUIDANCE2 online server (http://ww.guidance.tau.ac.il, accessed on 8 December 2021) [46]. Phylogenetic inferences were based on Bayesian analysis via CIPRES online server (https://www.phylo.org/index.php/, accessed on 8 December 2021). The best-fit model was determined using jModeltest v2.1.6 via CIPRES online server (https://www.phylo.org/index.php/, accessed on 8 December 2021) [47]. The phylogenetic analyses were performed using the obtained sequences from the 16S rRNA, 23S rRNA, *RNAse P*, and *dnaK*-based PCR protocols. All the sequences obtained from the present work were submitted to the GenBank database.

### 2.7. Genetic Diversity Assessment

The genetic diversity assessment was performed using only the obtained 16S rRNA sequences. In order to calculate nucleotide diversity (π), polymorphism level (haplotype diversity—[dh]), number of haplotypes (h), and the average number of nucleotide differences (K) among the sequences obtained, the DnaSP program v5 (http://www.ub.edu/dnasp/, accessed on 21 December 2021) [48] was used. The Genotype network was constructed in PopART (http://popart.otago.ac.nz, accessed on 11 December 2021), using the TCS inference method [49]. GPS coordinates collected for each sampling location were used to develop a map representing the genotype distribution using PopART software. Additionally, a distance-based analysis was performed using SplitsTree v4.14.6 (University of Tubingen, Tubingen, Germany) [50] to investigate the genetic relationship among hemoplasma genotypes detected in the present study and those previously deposited in GenBank. The pairwise distance matrix from the alignment of the 16S rRNA sequences detected in tapirs in the current study was calculated using the *p*-distance model and included Transitions + Transversions substitutions with uniform rates. Analysis was performed using MEGAX software (https://www.megasoftware.net, accessed on 17 December 2021) [51,52]. Data were transferred to a Microsoft Excel 2016 sheet to construct the heat map according to the rates obtained on the pairwise distance matrix.

### 2.8. Statistical Analysis

The chi-square test was used to determine associations between variables (gender, location, and age) and the outcomes (positive or negative PCR results for hemoplasmas). Odds ratio (OR), 95% confidence interval, and *p*-values were calculated for each variable. Results considered significantly different when *p* < 0.05. Data were compiled and analyzed in Epi Info™ software (v7.1.5, Centers for Disease Control and Prevention, Atlanta, GA, USA).

## 3. Results

### 3.1. PCR for Mammals’ Endogenous Genes

Out of 125 blood samples tested for the presence of the gapdh gene fragment, 9 (9/125; 07.20% CI: 03.83–13.12%) did not amplify fragments of the expected size and were submitted to the irpb-based PCR protocol. Out of 9 analyzed samples, 3 (3/125; 02.40%; CI: 00.82–06.82%) did not successfully amplify fragments of the expected size and were excluded from the following analyses. All excluded samples were obtained from road-killed tapirs.

### 3.2. PCR Assays for Hemoplasmas

From 122 analyzed samples, 41 (41/122; 33.61% CI: 25.84–42.38%) presented bands of expected size on electrophoresis after being processed by the partial 16S rRNA-based PCR protocol. The positive samples were obtained from 34 living animals and two road-killed animals. Out of the 34 positive living animals, 20 were identified as males (20/34; 58.82% CI: 42.22–73.63%) and 14 were identified as females (14/34; 41.18% CI: 26.37–57.78%). Moreover, 30 animals were from the Pantanal (30/34; 88.24% CI: 73.38–95.33%) and 4 from the Cerrado (04/34; 11.76% CI: 04.67–26.62%). Fifteen (15/43; 44.12% CI: 28.88–60.55%) were adults and 19 (19/34; 55.88% CI: 39.45–71.12%) sub-adults. Some animals (*n* = 3) presented positive samples in more than one sampling, totalizing seven positive samples (mean value: 2.3 positive samples/tapir) from the same animals. The two samples from road-killed animals that yielded positive results for the partial 16S rRNA gene were obtained from a sub-adult female and an adult male. In total, 35.29% (36/102; CI: 26.71–44.95%) tapirs (living or road-killed) presented positive results for hemotropic *Mycoplasma* sp. Results regarding positive samples, sampling dates, age/gender/location from tapirs, and GenBank accession numbers were summarized in Table 2.

In BLASTn analysis, 16S rRNA sequences obtained from 28 samples presented identity rates ranging from 97.20–98.20% with sequences of ‘*Candidatus* Mycoplasma haematoparvum’ from dogs from the USA and Italy (MH094850, AY383241), with query cover values of 99–100% and an E-value of 0.0. Meanwhile, two partial 16S rRNA gene sequences presented identity rates of 94.99% with ‘*Candidatus* Mycoplasma haematobos’ from cattle from Cuba (MG948628) with 100% of query cover and an E-value of 0.0. Regarding partial 23S rRNA analysis, 18 samples (18/41; 43.90% CI: 29.89–58.96%) presented bands of the expected size. From these, seven samples that presented high intensity and unique bands on agarose gel electrophoresis were submitted to sequencing and deposited in the GenBank database (OM022254-OM022260). When amplifying a fragment of approximately 165 bp from the *RNAse P* gene, 12 samples (12/41; 29.27%; 17.61–44.48%) presented bands of the expected size. From this total, two samples were successfully sequenced and deposited in the GenBank database (OM317758-OM317759). Finally, two samples (2/41; 4.88%; CI: 1.35–16.14%) submitted to *dnaK*-based PCR protocol presented expected band sizes in agarose gel electrophoresis and one was successfully sequenced and deposited in the GenBank database (OM339521) (Table 2). Only one sample (ID: MIA-P) presented sequences for all four targeted genes. The BLASTn analysis results for each sequence obtained by amplifying 23S rRNA, *RNAse P*, and *dnaK* genes in the present study are shown in Table 3, Table 4 and Table 5.

### 3.3. Phylogenetic Inference

**Figure 1:**
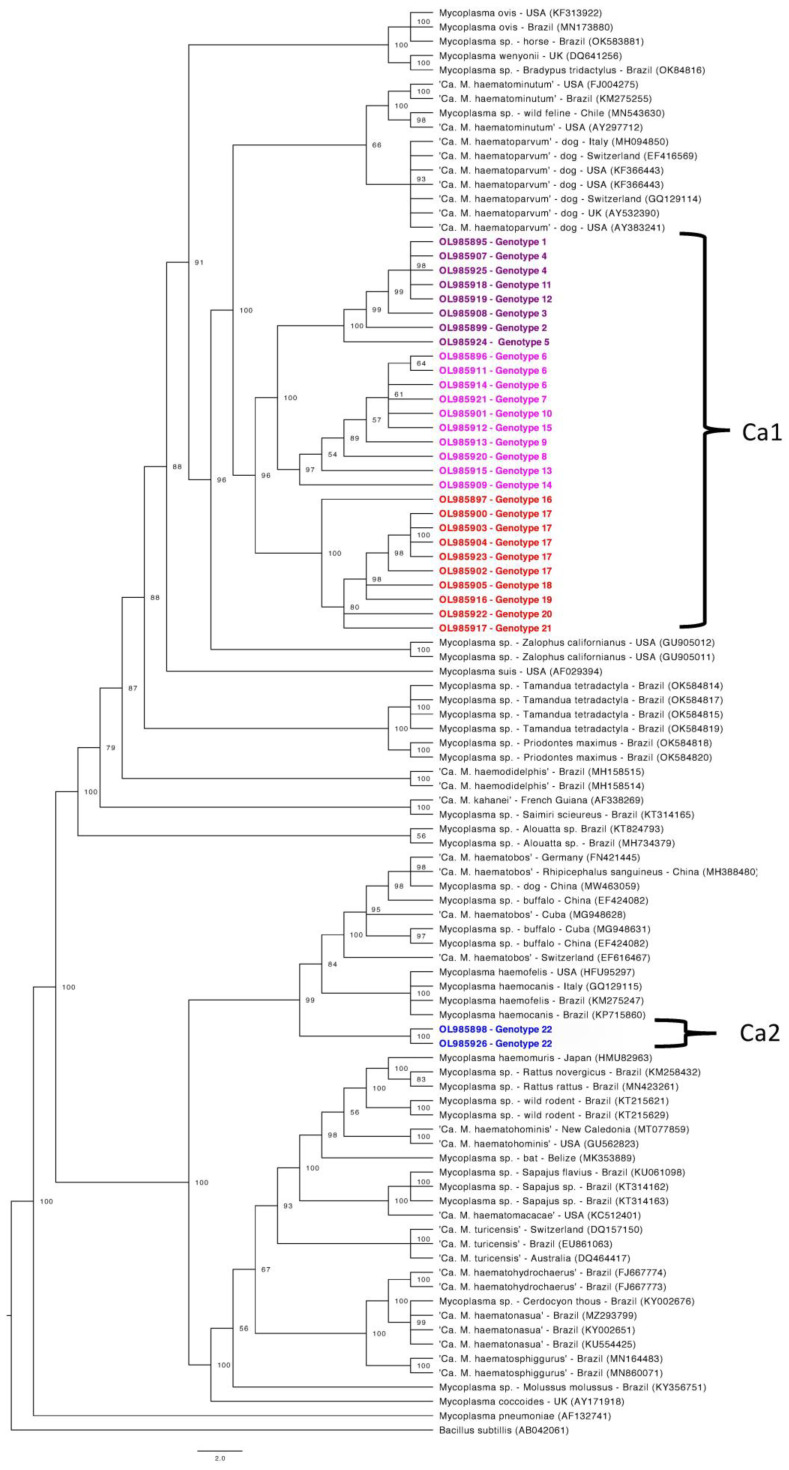
Phylogenetic tree based on partial 16S rRNA gene fragments of *Mycoplasma* sp. Tree was constructed by Bayesian Inference and a sequence from *Bacillus subtillis* (AB042061) was used as outgroup. Sequences obtained in the present study (Ca1 and Ca2) are highlighted in colors: Ca1 are highlighted in purple, pink and red colors, differentiating each subclade formed. Ca2 is highlighted in blue.

**Figure 2:**
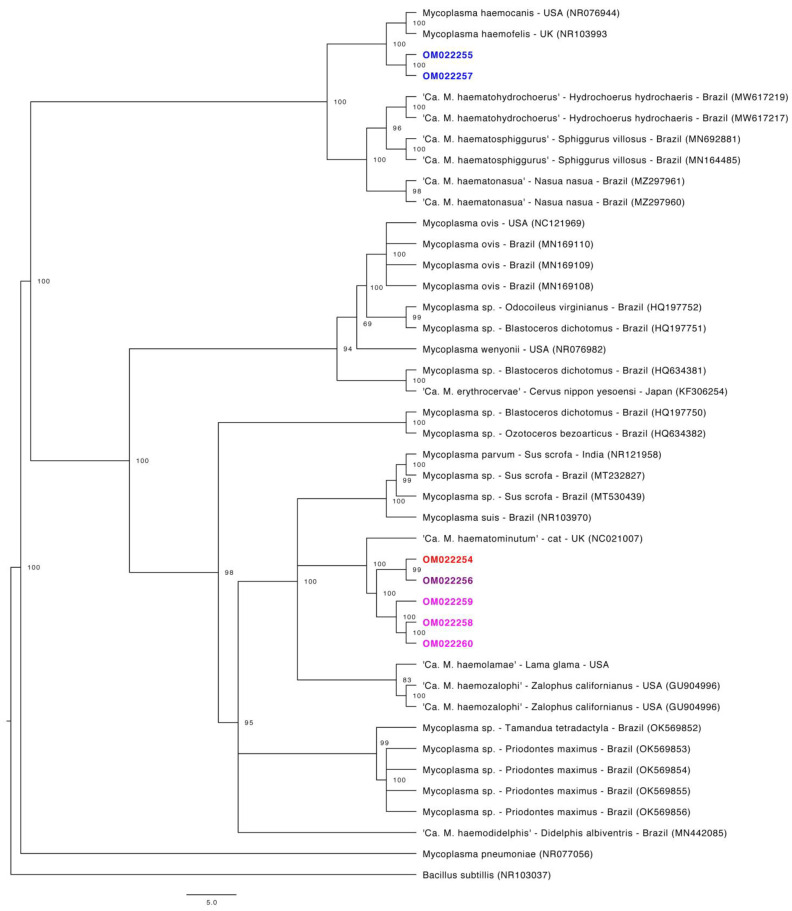
Phylogenetic tree based on partial 23S rRNA gene fragments of *Mycoplasma* sp. Tree was constructed by Bayesian Inference and a sequence from *Bacillus subtillis* (NR103037) was used as outgroup. Sequences obtained in the present study are highlighted in colors: sequences that fit in Ca1 by the 16S rRNa phylogeny are highlighted in purple, pink and red colors. Sequences that fit in Ca2 by the 16S rRNA phylogeny are highlighted in blue.

**Figure 3:**
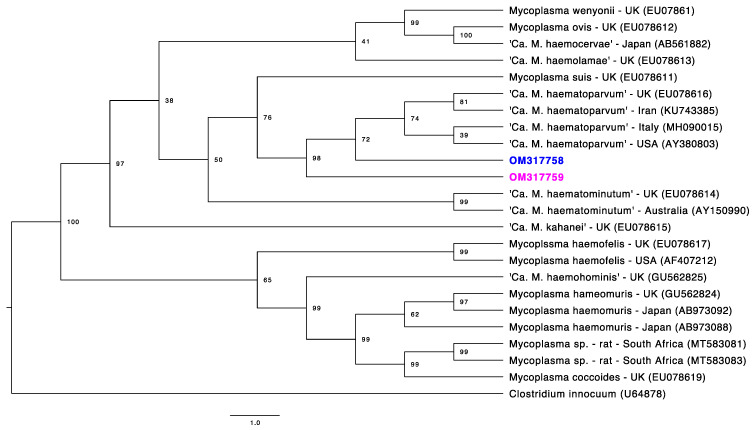
Phylogenetic tree based on partial *RNAse P* gene fragments of *Mycoplasma* sp. Tree was constructed by Bayesian Inference and a sequence from *Clostridium innocuum* (U64878) was used as outgroup. Sequences obtained in the present study are highlighted in colors: the sequence that fit in Ca1 by the 16S rRNA phylogeny is highlighted in pink. The sequence that fit in Ca2 by the 16S rRNA phylogeny is highlighted in blue.

**Figure 4:**
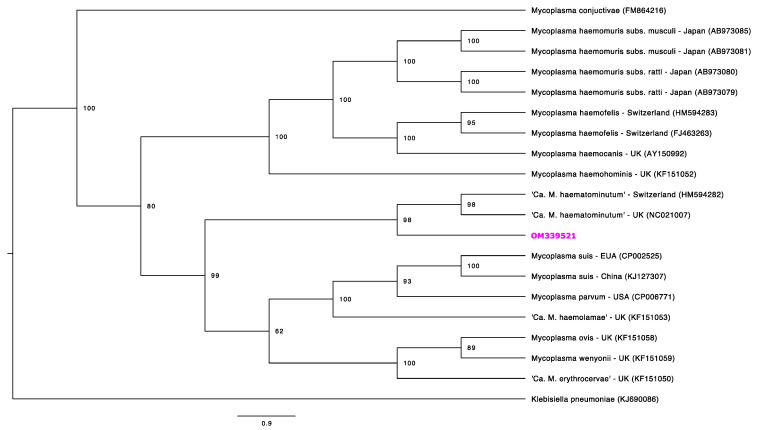
Phylogenetic tree based on partial *dnaK* gene fragments of *Mycoplasma* sp. Tree was constructed by Bayesian Inference and a sequence from *Klebisiella pneumonia* (KJ690086) was used as outgroup. Sequences obtained in the present study is highlighted in pink, once it fits in Ca1 by the 16S rRNA phylogeny.

Phylogenetic trees were constructed for four partial gene fragments by Bayesian inference using 10^7^ generations of MCMC (Monte Carlo Markov Chains) with two independent runs and 10% of burn-in (Figure 1, Figure 2, Figure 3 and Figure 4). For the partial 16S rRNA gene analysis (Figure 1), a total size alignment of 909 bp was constructed using 99 homolog sequences and one outgroup (AB042061).

The best-fit model for this gene analysis was determined as F81+G. For the partial 23S rRNA gene analysis (Figure 2), an alignment with a total size of 720 bp was constructed using 41 homolog sequences and one outgroup (NR103037). The best-fit model for this gene analysis was determined as F81+G. For the partial *RNAse P* gene analysis (Figure 3), a total size alignment of 230 bp was constructed using 23 homolog sequences and one outgroup (U64878). The best-fit model for this gene analysis was determined as F81+I+G. For the partial *dnaK* gene analysis (Figure 4) a total size alignment of 622 bp was constructed using 19 homolog sequences and one outgroup (KJ690086). The best-fit model for this gene analysis was determined as F81+G.

Regarding the 16S rRNA phylogenetic analysis, our sequences were divided into two different major clades. The first clade was also sub-divided into three minor clades and positioned in the “*Mycoplasma suis* group”, whereas the second clade comprised of only three sequences and was distantly positioned in the “*Mycoplasma haemofelis* group”. These results suggest the occurrence of two distinct species occurring in sampled tapirs. In order to facilitate the understanding from now on these two clades will be identified as Ca1 and Ca2, respectively. Sequences that fitted in Ca1 on partial 16S rRNA-based phylogeny followed a similar pattern in other genes phylogenies, except for the *RNAse P* gene. In the phylogenetic tree based on the *RNAse P* gene, both obtained sequences were positioned in the “*M. suis* group”, albeit each one was positioned in a different group on 16S rRNA phylogeny. Moreover, one sample (ID: AA-P) that was positioned in Ca1 on 16S rRNA-based phylogeny was found in Ca2 in the phylogenetic inference based on the partial 23S rRNA.

### 3.4. Genetic Diversity Assessment

**Figure 5:**
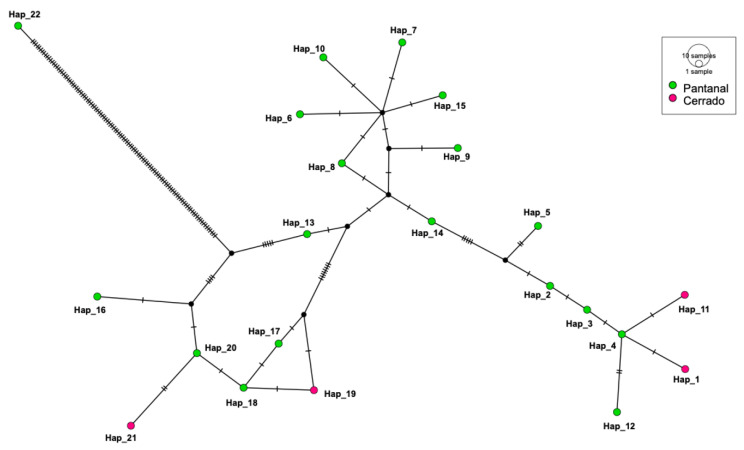
Genotype diversity among 16S rRNA gene sequences detected herein. Analysis was made using DnaSP6. Inference and graphic representation were made by TCS network method on PopART software. Genotypes in blue were obtained from samples from tapirs in Pantanal regions meanwhile genotypes in green were obtained from samples from tapirs in Cerrado regions.

**Figure 6:**
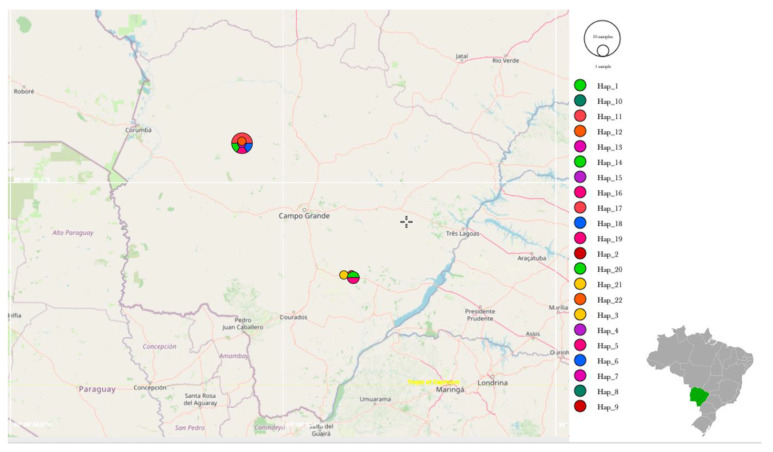
Representation of the genotype distribution along all sampling areas. Map was constructed using PopART software based on GPS coordinates data of each sampling.

**Figure 7:**
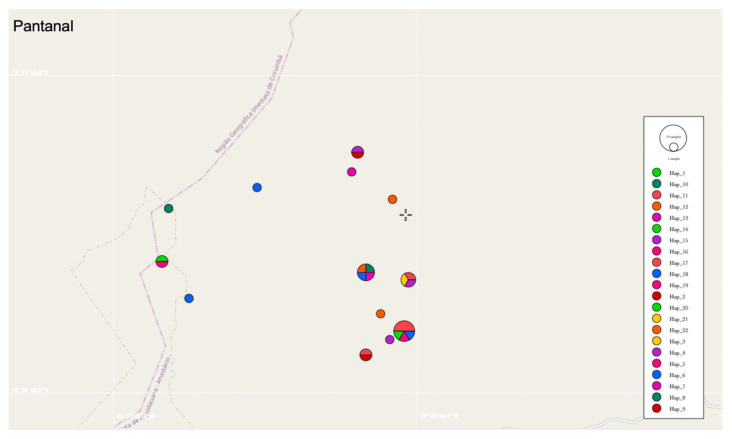
Representation of the genotype distribution of Pantanal biome areas. Map was constructed using PopART software based on GPS coordinates data of each sampling.

**Figure 8:**
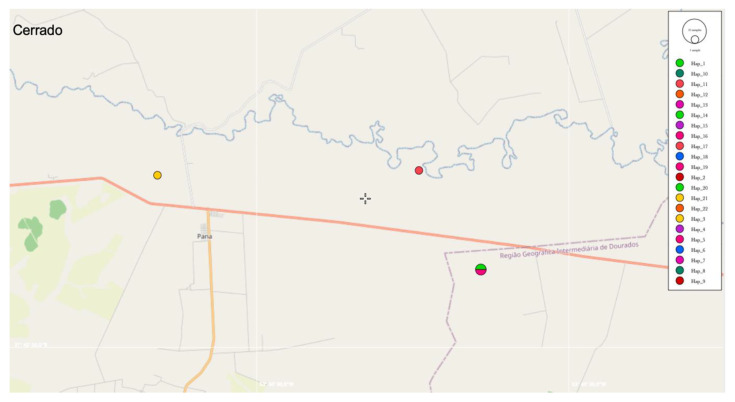
Representation of the genotype distribution of Cerrado biome areas. Map was constructed using PopART software based on GPS coordinates data of each sampling.

Regarding the 16S rRNA genotype analysis, 22 different genotypes were identified among 30 sequences (Figure 5). Values of nucleotide diversity (π), polymorphism level (haplotype diversity—[dh]), number of haplotypes (h), average number of nucleotide differences (K) among the sequences obtained, and the number of mutations between sequences from Ca1 and Ca2 are described in Table 6. 16S rRNA genotypes and sampling location of each obtained sequence are shown in Supplementary Table S1. The map representing genotype distributions along the sampling area reinforced the geographical distance between genotypes identified in Pantanal and Cerrado areas (Figure 6, Figure 7 and Figure 8).

### 3.5. Distance Analysis by SplitsTree

**Figure 9:**
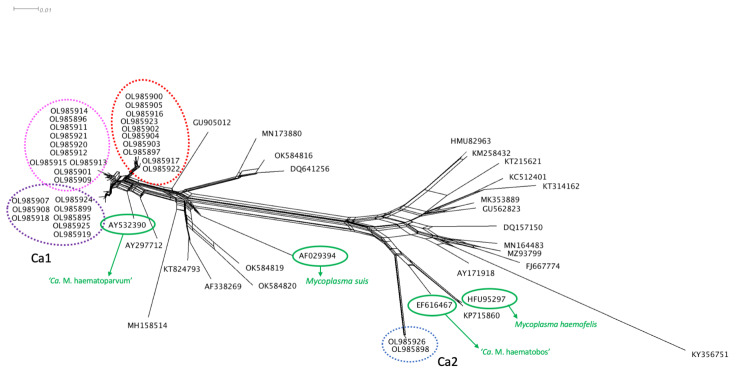
Distance analysis of 16S rRNA fragments from different *Mycoplasma* species was made using SplitsTree v4.14.6 software. Sequences from Ca1 and Ca2 are indicated in the tree. Regarding Ca1 sequences, colors from the Splitstree graph match with the subclades highlighted in 16S rRNA phylogeny (purple, pink and red). The Ca2 sequences are highlighted in blue. The species *M. suis* and *M. haemofelis* are highlighted in green. The species ‘*Ca*. M. haematoparvum’ and ‘*Ca*. M. haematobos’ were also highlighted in green to demonstrate their position compared to Ca1 and Ca2.

The distance analysis performed by the neighbor-joining method and displayed by SplitsTree v4.14.6 demonstrated that Ca1 and Ca2 sequences were distinctly disposed among other hemotropic *Mycoplasma* species and Candidatus (Figure 9).

### 3.6. Distance Matrix Analysis

**Figure 10:**
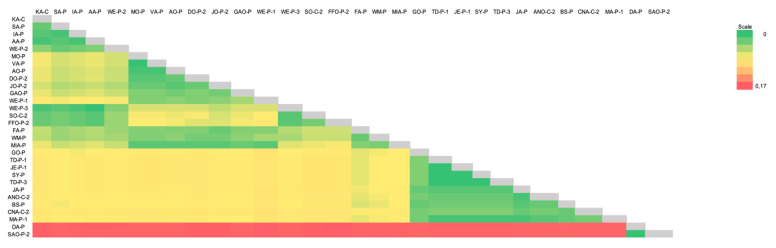
A heatmap constructed using the distance matrix based on *p*-value between all sequences obtained herein. The scale on the right demonstrates the color shade transition between obtained values.

The heat map with the distance matrix from all partial 16S rRNA sequences detected herein corroborated with other achieved results from the present study and demonstrated a marked difference between sequences from Ca1 and Ca2 (Figure 10). The minimum and maximum divergence percentage rates found for all the sequences that fit in Ca1 were 0.0% and 2.92%, respectively. When comparing to the phylogenetic closest sequences (FJ004275, KM275255, MN543630, AY297712, MH094850, EF416569, GQ129112, KF366443, GQ129114, AY532390, AY383241), the minimum and maximum divergence found between Ca1 members and those sequences was 1.52% and 3.27%, respectively. When comparing the Ca1 members and Ca2 members, the minimum and maximum divergence found was 15.23% and 16.22%, respectively. The minimum and maximum divergence percentage rates found for all members from Ca2 were 0.0%. When comparing to the phylogenetic closest sequences (FN421445, MG948628, MG948631, MH388480, MW463059, EF424082, EF616467, EF460765, HFU95297, GQ129115, KM275247, KP715860), the minimum and maximum divergence found between Ca2 members and those sequences was 5.12% and 7.23%, respectively. The distance matrix is available in Supplementary Table S2.

### 3.7. Statistical Analysis

Statistical analysis results including OR (odds ratio) and *p*-value are summarized in Table 7. Regarding the analysis between location and positivity for hemoplasmas, the number of positive tapirs sampled in Pantanal wetlands was statistically higher when compared to those sampled in the Cerrado region (*p*-value = 0.0001). The other analyzed variables (gender and age) did not present statistically significant differences for this outcome (*p*-value > 0.05). The OR value demonstrated that tapirs from Pantanal were 5.64 times more likely to present positive results for the PCR protocol targeting the partial Mycoplasma spp. 16S rRNA gene used in the present study when compared to tapirs sampled in Cerrado.

## 4. Discussion

We described herein the occurrence of at least two genetically distinct hemoplasma species occurring in free-ranging tapirs. Reports of *Candidatus* and novel species of these bacteria in Brazilian wild fauna are becoming common due to the growing use of molecular and genetic analysis tools. Based mainly on the partial 16S rRNA and 23S rRNA genes amplification, novel species of hemotropic *Mycoplasma* spp. infecting capybaras [22], hairy dwarf porcupines [23], opossums [24], and coatis [25] have been proposed. When analyzing the topologies found by partial 16S rRNA gene phylogeny from these reports, sequences from each novel *Candidatus* species were positioned separately in clades inside the “*Mycoplasma haemofelis* group”, with satisfactory bootstrap values for species separation. In the present work, we detected different sequences from tapirs that fit in both the “*Mycoplasma haemofelis* group” (Ca2) and “*Mycoplasma suis* group” (Ca1) by partial 16S rRNA, 23S rRNA, and *dnaK* genes-based phylogeny, which was unprecedented for putative novel species of hemoplasmas reported in wild hosts from Brazil.

The 16S rRNA is considered the “gold standard” target gene for PCR assays aiming at detecting and identifying hemoplasmas and different pairs of primers have been used for its purpose [15]. Although this gene may be considered highly conserved, the 16S rRNA gene from bacterial species may exhibit considerable variations even on supposed conserved regions [53], which allow species differentiation. In the present study, all partial 16S rRNA sequences obtained for the Ca1 genotypes presented a similarity range of 97.20–98.20% with ‘*Candidatus* Mycoplasma haematoparvum’ detected in dogs from Italy and the USA (MH094850, AY383241) by BLASTn analysis. Meanwhile, the Ca2 genotypes demonstrated a similarity range of 94.99% with ‘*Candidatus* Mycoplasma haematobos’ detected in cattle from Cuba (MG948631). The *p*-distance values obtained by the distance matrix analysis showed that Ca1 members presented divergence rates of 0.0–2.92%, whereas the same comparison using the closest phylogenetic group and members of Ca1 presented rates of 1.52–3.27%. When using the 16S rRNA gene, a similarity rate of at least <97% is expected for bacteria belonging to different species. However, divergence rates <3% do not necessarily indicate that sequences belong to the same species [54]. Once phylogenetic analyses based on four molecular markers (16S rRNA, 23S rRNA, *RNAse P*, and *dnaK)* and 16S-based distance analyses strongly supported the differentiation between Ca1 and the closest hemoplasma species (‘*Ca*. M. haematoparvum’), it is likely that Ca1 represents a genetically distinct group of hemoplasmas.

The use of different genes for the molecular characterization of hemoplasmas is reported. Amplification of partial 23S rRNA gene was reported in phylogenetic studies of hemoplasmas identified in captive cervids from southern Brazil [55] and for the phylogenetic study of *Mycoplasma ovis* infecting sheep from the same region [18]. Recently, this gene has also been used for the characterization of novel *Candidatus* species of hemoplasmas from wild animals in Brazil [22,23,24,25]. In the present study, partial 23S rRNA sequences that fit in Ca1 presented similarity percentages ranging from 89.59–91.50% with a sequence of ‘*Candidatus* Mycoplasma haematominutum’ from the UK (HE613254). Meanwhile, 23S rRNA sequences that fit in Ca2 presented similarity ranges of 90.20–90.57% with a sequence of *Mycoplasma haemofelis* from the USA (NR103993). In fact, putative novel hemoplasma species may present lower similarity rates in BLASTn analysis for 23S rRNA sequences when compared to 16S rRNA sequences [22,55,56]. These differences may be explained by the fact that, although the 23S rRNA is considered as phylogenetically conserved as 16S rRNA, it presents a higher degree of sequence variability [57].

The *RNAse P* (*rnpB*) gene codifies the RNA subunit of endoribonuclease P with a length of approximately 400 bp [58]. Although only a few numbers of studies used phylogenetic trees of hemoplasmas using fragments of the *RNAse P* gene, our phylogeny analysis using this gene agreed with these studies, showing a separation of species among the “*Mycoplasma haemofelis* group” and “*Mycoplasma suis*/‘*Ca*. M. haematominutum’ group” [16,58]. However, both sequences obtained in the present study (OM317758-OM317759) were positioned in the “*Mycoplasma haemofelis* group”, albeit these same two samples were positioned separately in the “*Mycoplasma haemofelis* group” and “*Mycoplasma suis* group” in all other analyzed targeted genes. Although it is expected that phylogenies using *RNAse P* fragments of hemoplasmas may present similar topologies to those using 16S rRNA [58], the fragments obtained herein are very short (~100 bp), precluding robust species differentiation.

Amplification and subsequent phylogenetic analysis of the *dnaK* gene have been demonstrated to be a useful tool for the separation of hemoplasmas from other Mollicute species [59] and also for the separation of subspecies of hemoplasmas [60]. This gene is responsible for coding a chaperon protein (heat shock protein 70) and is considered a great genetic marker for species differentiation, since it may present more variable regions within the sequences when compared to ribosomal fragments [59]. Unfortunately, only one sequence from the present study was successfully sequenced using the *dnaK* (OM339521) PCR protocol, and comparison with other sequences obtained from tapirs was not possible. However, this single sequence followed a similar topology pattern of 16S rRNA and 23S rRNA phylogenies, since it fitted on Ca1 in the “*Mycoplasma suis* group”, reinforcing the phylogenetic position found for this putative novel hemoplasma species.

A high diversity of genotypes (*n* = 22) was found in the present work by analysis of 30 partial 16S rRNA sequences of hemoplasmas obtained from tapirs’ samples. Richness on genotype diversity was also reported for hemoplasmas detected in bats from Brazil [56]. Although the genotypes found in tapirs from the Pantanal diverged from those found in tapirs from the Cerrado, a genetic proximity between these genotypes was demonstrated on the genotype network analysis. The occurrence of more than one genotype in the same region infecting wild hosts has already been reported in bats from Brazil [56]. Interestingly, one animal that was sampled at three different times (IDs: WE-P-1, WE-P-2, and WE-P-3) presented three different genotypes according to the time of sampling. Moreover, one animal (ID: AA-P), that was sampled only once, was positioned in Ca1 on 16S rRNA-based phylogeny and in Ca2 in the phylogenetic inference based on the partial 23S rRNA. These data suggest that tapirs may be susceptible to co-infections with different hemoplasma genotypes or species, with these co-infections occurring simultaneously or at different times.

Some animals that were sampled more than once presented positive samples in more than one sampling (IDs: MA-P-1, MA-P-2, JE-P-1, JE-P-2, WEP-1, WEP-2, WE-P3). Meanwhile, some animals that also were sampled more than once presented positive results for one sample only (IDs: TD-P-1, VA-P-1, ANO-C-2, CNA-C-2, SO-C-2, FFO-P-2, JO-P-2, DO-P-2, TD-P-3, SAO-P-2). These results may suggest either that tapirs may be maintained as chronically infected hosts or that hemoplasma bacteremia may be too low, precluding molecular detection. Chronic infection by hemoplasmas has been commonly reported in domestic species, such as pigs [61], cattle [62], and cats [63,64]. Although it is not possible to extend these findings for wildlife, chronic infection by hemoplasmas is appointed as a cause of unthriftiness in newborn piglets [65] and lower calf birth weight in cattle [62] which may raise a red flag in the context of species conservation.

Herein, gender and age were not associated with hemoplasma infection in the sampled tapirs. Although tapirs from Cerrado presented more health abnormalities when compared to tapirs from other biomes [65], we found that animals sampled in the Pantanal were 5.64 times more prone to be infected by hemoplasmas when compared to tapirs sampled in the Cerrado. In cats, population density may be associated as a risk factor for hemoplasma infection [66]. Some Pantanal sites and habitats were already reported as able to sustain high densities of tapir populations when these animals are not exposed to anthropic actions [67].

Tapirs from both Cerrado and Pantanal regions were found parasitized by *Amblyomma* spp. and *Rhipicephalus microplus* ticks. Although information regarding the transmission of hemoplasmas by ticks and other vectors is still lacking, *R. microplus* ticks were appointed as capable of transmitting ‘*Ca*. M. haematobos’ for egg and larval stages and also to transmit this hemoplasma species for mice [68]. Future studies aiming at investigating the role of ticks in the transmission of hemoplasmas among tapirs are needed.

*Mycoplasma haemofelis* and ‘*Ca*. M. haematominutum’ DNA was detected in saliva and salivary glands of infected cats, suggesting that social interactions, such as aggression, may be related to hemoplasma infections [69]. Lowland tapirs were considered to be as solitary and show tolerance to other individuals not influenced by kinship [70]. More studies are necessary to elucidate the transmission routes for hemoplasmas among free-ranging lowland tapirs in Brazil.

The occurrence of anemia due to hemoplasma infection has been common in cats [15,19,71,72], pigs [61], cattle [73], sheep [74,75], and splenectomized dogs [76]. Regarding hemoplasma infection in wild animals, the occurrence of anemia has already been reported in reindeers (*Rangifer tarandus*) [77], non-human primates (*Sapajus flavius)* [17], and guignas (*Leopardus guigna*) [78]. Considering the occurrence of 35.29% (36/102; CI: 26.71–44.95%) infected individuals found in the present study, health assessment of infected animals may be useful to understand if hemoplasma-induced anemia is a threat for the largest land mammal species from Brazil.

Besides the assessment, for the first time, of the occurrence of hemotropic *Mycoplasma* among tapirs in two distinct Brazilian biomes, the present work showed high genetic diversity, and, at least, two genetically distinct hemoplasma species infecting these mammals. Accordingly, the obtained results reinforce the need for multi-locus and large-scale sequencing aiming at unraveling accurately the genetic diversity of hemoplasmas in wild animals.

## 5. Conclusions

At least two genetically distinct species of hemotropic *Mycoplasma* spp. occurs in free-ranging *T. terrestris* from the Pantanal and Cerrado regions in Brazil. The occurrence of hemoplasmas did not differ according to the gender or age of the sampled tapirs. Animals sampled in the Pantanal may be at a higher risk of becoming infected by hemoplasma when compared to those in the Cerrado. We propose that the two genetically divergent species found infecting tapirs from the present study represent putative novel *Candidatus* species and the names ‘*Candidatus* Mycoplasma haematoterrestris’ and ‘*Candidatus* Mycoplasma haematotapirus’ are proposed for the species found in Ca1 and Ca2, respectively.

## Figures and Tables

**Table 1:** Target genes, primers, thermal conditions, and reagent protocol used in the PCR assays for hemoplasmas based on the 16S rRNA, 23S rRNA, *RNAse P*, and *dnaK* genes.

Target Gene	Primer Sequences	Thermal Conditions	Reagents Volumes and Concentration	Fragment Size	Primers Reference
16S rRNA	1st round: 5′-AGAGTTTGATCCTGGCTCAG-3′ 5′-ACCGCAGCTGCTGGCACATA-3′ 2nd round: 5′-ATATTCCTACGGGAAGCAGC-3′ 5′-ACCGCAGCTGCTGGCACATA-3′	95 °C for 5 min, followed by 35 cycles of denaturation at 95 °C for 30 s annealing at 57 °C for 30 s extension at 72 °C for 1 min, and final extension at 72 °C for 10 min for both rounds.	1st reaction: 2.5 μL from 10X Buffer, 0.75 μL from 50 mM MgCl_2_, 2 μL from 10 mM dNTP mix, 1 μL from each primer at 10 mM, 0.25 μL from 5 U/ μL Taq polymerase, 12.5 μL from ultrapurified water and 5 μL from template DNA. 2nd reaction: Ultrapurified water (16.5 μL) and template DNA (1 μL) quantities changes.	~1107 bp	Harasawa et al., 2014; Di Cataldo et al., 2020
23S rRNA	5′-TGAGGGAAAGAGCCCAGAC-3′ 5′-GGACAGAATTTACCTGACAAG G-3′	94 °C for 3 min, followed by 35 cycles of denaturation at 94 °C for 30 s annealing at 54 °C for 30 s extension at 72 °C for 1 min, and final extension at 72 °C for 10 min.	2.5 μL from 10X Buffer, 0.75 μL from 50 mM MgCl_2_, 2 μL from 10 mM dNTP mix, 1 μL from each primer at 10 mM, 0.25 μL from 5 U/μL Taq polymerase, 12.5 μL from ultrapurified water and 5 μL from template DNA.	~800 bp	Mongruel et al., 2020
*RNAse P*	5′-GATKGTGYGAGYATATAA AAAATAAARCTCRAC-3′ 5′-GMGGRGTTTACCGCGTTTCAC-3′	95 °C for 2 min, followed by 50 cycles of denaturation at 94 °C for 30 s annealing at 59 °C for 30 s extension at 72 °C for 30 s and final extension at 72 °C for 1 min.	2.5 μL from 10X Buffer, 1.0 μL from 50 mM MgCl_2_, 2 μL from 10 mM dNTP mix, 1 μL from each primer at 10 mM, 0.25 μL from 5 U/μL Taq polymerase, 12.25 μL from ultrapurified water and 5 μL from template DNA.	~164 bp	Maggi et al., 2013
*dnaK*	5′-GGGTGGAGATGATTGAGA CCA-3′ 5′-GGGTGGAGATGATTGAGACCA-3′	95 °C for 5 min, followed by 45 cycles of denaturation at 95 °C for 20 s annealing at 55.5 °C for 30 s extension at 72 °C for 45 s and final extension at 72 °C for 7 min.	2.25 μL from 10X Buffer, 1.0 μL from 50 mM MgCl_2_, 2 μL from 10 mM dNTP mix, 1 μL from each primer at 10 mM, 0.15 μL from 5 U/μL Taq polymerase, 12.6 μL from ultrapurified water and 5 μL from template DNA.	~544 bp	Descloux et al., 2020

**Table 2:** Identification, sampling dates, locations, gender/age, and Genbank accession number for each partial 16S rRNA-positive animals.

Animal ID	SamplingDate	Biome	Gender/Age	16S rRNA GenBank Accession Number	23S rRNA GenBank Accession Number	*RNAse P* GenBank Accession Number	*dnaK* GenBank Accession Number
KA-C	20 December 2016	Cerrado	Male/adult	OL985895	NS	Negative	Negative
MO-P	08 December 2014	Pantanal	Male/juvenile	OL985896	NS	Negative	Negative
MA-P-1 **	28 July 2013	Pantanal	Female/adult	OL985902	NS	Negative	Negative
GO-P	13 July 2014	Pantanal	Male/sub-adult	OL985897	NS	Negative	Negative
JE-P-1 **	06 December 2017	Pantanal	Male/sub-adult	OL985903	NS	Negative	Negative
TD-P-1 *	16 November 2015	Pantanal	Male/sub-adult	OL985900	OM022254	NS	Negative
SY-P	28 October 2017	Pantanal	Female/adult	OL985904	NS	Negative	Negative
RA-P	04 May 2014	Pantanal	Female/juvenile	NS	Negative	Negative	Negative
CO-P	31 October 218	Pantanal	Male/sub-adult	NS	Negative	Negative	Negative
CJO-P	06 July 2014	Pantanal	Male/adult	NS	Negative	Negative	Negative
CIO-P	23 July 2013	Pantanal	Male/sub-adult	NS	NS	Negative	Negative
JA-P	05 May 2014	Pantanal	Female/juvenile	OL985905	NS	Negative	Negative
LA-P	29 August 2017	Pantanal	Female/juvenile	NS	Negative	Negative	Negative
AA-P	22 October 2018	Pantanal	Male/sub-adult	OL985907	OM022255	Negative	Negative
MU-P	22 October 2013	Pantanal	Female/adult	NS	Negative	Negative	Negative
IA-P	10 December 2017	Pantanal	Male/adult	OL985908	Negative	Negative	Negative
WM-P	05 December 2014	Pantanal	Male/juvenile	OL985909	Negative	NS	Negative
SA-P	05 December 2017	Pantanal	Female/juvenile	OL985899	OM022256	NS	Negative
DA-P	25 August 2018	Pantanal	Female/sub-adult	OL985898	OM022257	OM317758	Negative
RTA-P	10 May 2015	Pantanal	Female/adult	NS	Negative	NS	Negative
WE-P-1 **	24 November 2015	Pantanal	Female/sub-adult	OL985901	Negative	NS	Negative
VA-P-1 *	01 September 2018	Pantanal	Female/sub-adult	OL985911	Negative	Negative	Negative
MIA-P	25 June 2018	Pantanal	Female/sub-adult	OL985912	OM022258	OM317759	OM339521
GAO-P	19 June 2018	Pantanal	Male/adult	OL985913	Negative	NS	Negative
AO-P	08 June 2016	Pantanal	Male/juvenile	OL985914	OM022259	NS	Negative
FA-P	18 June 2018	Pantanal	Female/adult	OL985915	Negative	NS	Negative
MA-P-2 **	19 May 2015	Pantanal	Female/adult	NS	Negative	Negative	Negative
ANO-C-2 *	28 June 2017	Cerrado	Male/adult	OL985916	Negative	NS	Negative
CNA-C-2 *	19 September 2018	Cerrado	Female/adult	OL985917	Negative	NS	NS
SO-C-2 *	09 February 2017	Cerrado	Male/adult	OL985918	Negative	Negative	Negative
FFO-P-2 *	23 August 2017	Pantanal	Male/adult	OL985919	Negative	Negative	Negative
JO-P-2 *	20 August 2016	Pantanal	Male/sub-adult	OL985920	Negative	Negative	Negative
DO-P-2 *	25 June 2018	Pantanal	Male/sub-adult	OL985921	OM022260	Negative	Negative
JE-P-2 **	09 June 2016	Pantanal	Male/sub-adult	NS	Negative	Negative	Negative
BS-P	11 June 2016	Pantanal	Male/sub-adult	OL985922	Negative	Negative	Negative
TD-P-3 *	18 June 2016	Pantanal	Male/sub-adult	OL985923	NS	Negative	Negative
WE-P-2 **	16 June 2016	Pantanal	Female/sub-adult	OL985924	NS	Negative	Negative
WE-P-3 **	15 December 2016	Pantanal	Female/sub-adult	OL985925	NS	Negative	Negative
SAO-P-2 *	20 October 2013	Pantanal	Male/adult	OL985926	Negative	Negative	Negative
NEC09-C	09 April 2016	Cerrado	Female/sub-adult	NS	Negative	Negative	Negative
NEC18-C	29 July 2016	Cerrado	Male/adult	NS	Negative	Negative	Negative

* Animal with more than one sample collected. Did not yield positive results for partial 16S rRNA in all samples. ** Animal with more than one sample collected. Positive results for partial 16S rRNA in all samples. NS = Sequence presented expected size bands on electrophoresis but was not successfully sequenced.

**Table 3:** BLASTn analysis of each partial hemoplasma 23S rRNA sequence obtained from free-ranging tapirs from the Brazilian biomes of Cerrado and Pantanal.

Animal ID	23S rRNA GenBank Accession Number	BLASTn Best Hit	Host	Country	Query Cover (%)	E-Value	Identity (%)	Best Hit GenBank Accession Number
TD-P-1	OM022254	‘*Candidatus* Mycoplasma haematominutum’	*Felis catus*	England	100%	0.0	91.50%	HE613254
AA-P	OM022255	*Mycoplasma haemofelis*	*Felis catus*	England	99%	0.0	90.57%	NR103993
SA-P	OM022256	‘*Candidatus* Mycoplasma haematominutum’	*Felis catus*	England	99%	0.0	91.13%	HE613254
DA-P	OM022257	*Mycoplasma haemofelis*	*Felis catus*	England	100%	0.0	90.21%	NR103993
MIA-P	OM022258	‘*Candidatus* Mycoplasma haematominutum’	*Felis catus*	England	99%	0.0	89.90%	HE613254
AO-P	OM022259	‘*Candidatus* Mycoplasma haematominutum’	*Felis catus*	England	100%	0.0	90.36%	HE613254
DO-P-2	OM022260	‘*Candidatus* Mycoplasma haematominutum’	*Felis catus*	England	100%	0.0	89.59%	HE613254

**Table 4:** BLASTn analysis of each partial hemoplasma *RNAse P* sequence obtained from free-ranging tapirs from the Brazilian biomes of Cerrado and Pantanal.

Animal ID	*RNAse P* GenBank Accession Number	BLASTn Best Hit	Host		Query Cover (%)	E-Value	Identity (%)	Best Hit GenBank Accession Number
DA-P	OM317758	‘*Candidatus* Mycoplasma haematoparvum’	*Canis lupus familiaris*	Italy	95%	1 × 10^−34^	96.88%	MH090015
MIA-P	OM317758	‘*Candidatus* Mycoplasma haematoparvum’	*Canis lupus familiaris*	Italy	98%	4 × 10^−29^	93.62%	MH090015

**Table 5:** BLASTn analysis of each partial hemoplasma *dnaK* sequence obtained from free-ranging tapirs from the Brazilian biomes of Cerrado and Pantanal.

Animal ID	23S rRNA GenBank Accession Number	BLASTn Best Hit	Host	Country	Query Cover (%)	E-Value	Identity (%)	Best Hit GenBank Accession Number
MIA-P	OM339521	*‘Candidatus* Mycoplasma erythrocervae’	Not informed	England	87%	5 × 10^−59^	78.74%	KF‘51050

**Table 6:** Values obtained regarding genotype diversity by DnaSP software and based on partial hemoplasma 16S rRNA sequences detected in tapirs from the present study.

Nucleotide Diversity (π)	Genotype Diversity (dh)	Number of Haplotypes (h)	Average Number of Nucleotide Differences between All Sequences (K)	Average Number of Nucleotide Differences between Ca1 and Ca2	Number of Fixed Differences between Ca1 and Ca2
0.03112	0.966	22	24.21149	121.036	112

**Table 7:** Statistical analysis comparing the occurrence of hemotropic *Mycoplasma* sp. in sampled tapirs and outcomes (gender, sampling location, and age).

		16S rRNA Hemotropic *Mycoplasma* spp. PCR				
Variable		+/*n*	(%)	OR	95% CI	*p*-Value
**Gender**	Male Female Total	21/53 15/49 36/102	39.62 30.61	1.488	0.65–3.37	0.1707
**Location**	Pantanal	30/61	48.18	5.645	2.075–15.36	**0.0001721**
	Cerrado Total	6/41 36/102	14.63			
**Age**	Sub-adult Adult Total	20/47 16/55 36/102	42.22 29.09	1.806	0.79–4.10	0.09529

+, Number of positive animals; *n*, number of samples; 95% CI, 95% confidence interval; OR, odd ratio. *p*-values < 0.05 were considered statically significant and were highlighted in bold.

## Data Availability

Not applicable.

## Supplementary Materials

The following supporting information can be downloaded at: https://www.mdpi.com/article/10.3390/microorganisms10030614/s1, Table S1: Hemoplasma-16S rRNA genotypes found in tapirs sampled in the present study according to sampling location.; Table S2: Distance matrix for partial 16S rRNA.
