# Correction: Mongruel et al. Expanding the Universe of Hemoplasmas: Multi-Locus Sequencing Reveals Putative Novel Hemoplasmas in Lowland Tapirs (*Tapirus terrestris*), the Largest Land Mammals in Brazil. *Microorganisms* 2022, *10*, 614

**DOI:** 10.3390/microorganisms12071326

**Published:** 2024-06-28

**Authors:** Anna Claudia Baumel Mongruel, Emília Patrícia Medici, Ariel da Costa Canena, Ana Cláudia Calchi, Rosangela Zacarias Machado, Marcos Rogério André

**Affiliations:** 1 Immunoparasitology Laboratory, Department of Pathology, Theriogenology, and One Health, School of Agricultural and Veterinary Sciences, São Paulo State University, UNESP, Jaboticabal 14884-900, SP, Brazil; annaclaudiamongruel@gmail.com (A.C.B.M.); ana.calchi@unesp.br (A.C.C.); rzacariasmachado@gmail.com (R.Z.M.); 2 Iniciativa Nacional para a Conservação da Anta Brasileira (INCAB), Instituto de Pesquisas Ecológicas (IPÊ), Campo Grande 79046-150, MS, Brazil; medici@ipe.org.br (E.P.M.); ariel.canena@gmail.com (A.d.C.C.); 3 Escola Superior de Conservação Ambiental e Sustentabilidade (ESCAS/IPÊ), Nazaré Paulista 12960-000, SP, Brazil; 4 Tapir Specialist Group (TSG), International Union for Conservation of Nature (IUCN SSC), Campo Grande 79046-150, MS, Brazil

In the original publication [1], there was a mistake in Table 1 and Table 7 as published. The corrected tables appear below.

In Table 1 for the 16S rRNA partial amplification, a different combination of the cited primers was used. Regarding the *dnaK* primer sequences, a formatting oversight resulted in identical representations for forward and reverse primers. 

**Table 1:** Target genes, primers, thermal conditions, and reagent protocol used in the PCR assays for hemoplasmas based on the 16S rRNA, 23S rRNA, *RNAse P*, and *dnaK* genes.

Target Gene	Primer Sequences	Thermal Conditions	Reagent Volumes and Concentrations	Fragment Size	Primers Reference
16S rRNA	1st round: 5′-AGAGTTTGATCCTGGCTCAG-3’ 5′-TACCTTGTTACGACTTAACT-3′ 2nd round: 5′-ATATTCCTACGGGAAGCAGC-3′ 5′-TACCTTGTTACGACTTAACT-3′	95 °C for 5 min, followed by 35 cycles of denaturation at 95 °C for 30 s, annealing at 57 °C for 30 s, extension at 72 °C for 1 min, and final extension at 72 °C for 10 min for both rounds.	1st reaction: 2.5 μL from 10X Buffer, 0.75 μL from 50 mM MgCl_2_, 2 μL from 10 mM dNTP mix, 1 μL from each primer at 10 mM, 0.25 μL from 5 U/μL Taq polymerase, 12.5 μL from ultrapurified water and 5 μL from template DNA. 2nd reaction: Ultrapurified water (16.5 μL) and template DNA (1 μL) quantity changes.	~1107 bp	Harasawa et al., 2014; Di Cataldo et al., 2020
23S rRNA	5′-TGAGGGAAAGAGCCCAGAC-3′ 5′-GGACAGAATTTACCTGACAAGG-3′	94 °C for 3 min, followed by 35 cycles of denaturation at 94 °C for 30 s, annealing at 54 °C for 30 s, extension at 72 °C for 1 min, and final extension at 72 °C for 10 min.	2.5 μL from 10X Buffer, 0.75 μL from 50 mM MgCl_2_, 2 μL from 10 mM dNTP mix, 1 μL from each primer at 10 mM, 0.25 μL from 5 U/μL Taq polymerase, 12.5 μL from ultrapurified water and 5 μL from template DNA.	~800 bp	Mongruel et al., 2020
*RNAseP*	5′-GATKGTGYGAGYATATAA AAAATAAARCTCRAC-3′ 5′-GMGGRGTTTACCGCGTTTCAC-3′	95 °C for 2 min, followed by 50 cycles of denaturation at 94 °C for 30 s, annealing at 59 °C for 30 s, extension at 72 °C for 30 s and final extension at 72 °C for 1 min.	2.5 μL from 10X Buffer, 1.0 μL from 50 mM MgCl_2_, 2 μL from 10 mM dNTP mix, 1 μL from each primer at 10 mM, 0.25 μL from 5 U/μL Taq polymerase, 12.25 μL from ultrapurified water and 5 μL from template DNA.	~164 bp	Maggi et al., 2013
*dnaK*	5′-GGGTGGAGATGATTGAGACCA-3’ 5′-AGCCACCCCTCCTAGAGTTT-3'	95 °C for 5 min, followed by 45 cycles of denaturation at 95 °C for 20 s, annealing at 55.5 °C for 30 s, extension at 72 °C for 45 s and final extension at 72 °C for 7 min.	2.25 μL from 10X Buffer, 1.0 μL from 50 mM MgCl_2_, 2 μL from 10 mM dNTP mix, 1 μL from each primer at 10 mM, 0.15 μL from 5 U/μL Taq polymerase, 12.6 μL from ultrapurified water and 5 μL from template DNA.	~544 bp	Descloux et al., 2020

In Table 7 the statistical analysis was calculated based on the total sampled animals. However, DNA samples from three animals failed to amplify both of the tested housekeeping genes and were consequently excluded from hemoplasma molecular screening, as explained in the Materials and Methods and Results sections. Although the significance of the tested variables did not change, the statistical analysis was corrected. The corrected table appears below.

**Table 7:** Statistical analysis comparing the occurrence of hemotropic *Mycoplasma* sp. in sampled tapirs and outcomes (gender, sampling location, and age).

		16S rRNA *Mycoplasma* spp. PCR				
Variable		+/n	(%)	OR	95% CI	*p*-Value
**Gender**	Male Female Total	21/50 15/49 36/99	42 30.61	1.641	0.71–3.75	0.1198
**Location**	Pantanal Cerrado Total	30/61 6/38 36/99	49.18 15.79	5.161	1.887–14.11	**0.0003915**
**Age**	Sub-adult Adult Total	20/46 16/53 36/99	43.48 30.19	1.779	0.77–4.06	0.08528

+, Number of positive animals; n, number of samples; 95% CI, 95% confidence interval; OR, odds ratio. *p*-values < 0.05 were considered statically significant and are highlighted in bold.

The authors state that the scientific conclusions are unaffected. This correction was approved by the Academic Editor. The original publication has also been updated.
